# Intestinal Bacteria as Powerful Trapping Lifeforms for the Elimination of Radioactive Cesium

**DOI:** 10.3389/fvets.2019.00070

**Published:** 2019-03-12

**Authors:** Kazuki Saito, Kengo Kuroda, Rie Suzuki, Yasushi Kino, Tsutomu Sekine, Hisashi Shinoda, Hideaki Yamashiro, Tomokazu Fukuda, Jin Kobayashi, Yasuyuki Abe, Junko Nishimura, Yusuke Urushihara, Hiroshi Yoneyama, Manabu Fukumoto, Emiko Isogai

**Affiliations:** ^1^Graduate School of Agricultural Science, Tohoku University, Sendai, Japan; ^2^Department of Chemistry, Tohoku University, Sendai, Japan; ^3^Center for the Advancement of Higher Education, Tohoku University, Sendai, Japan; ^4^Graduate School of Dentistry, Tohoku University, Sendai, Japan; ^5^Graduate School of Science and Technology, Niigata University, Niigata, Japan; ^6^Faculty of Science and Engineering, Iwate University, Morioka, Japan; ^7^School of Food, Agricultural and Environmental Sciences, Miyagi University, Sendai, Japan; ^8^Faculty of Life and Environmental Sciences, Prefectural University of Hiroshima, Hiroshima, Japan; ^9^Department of Biotechnology and Environmental Engineering, Faculty of Engineering, Hachinohe Institute of Technology, Hachinohe, Japan; ^10^Department of Radiation Biology, Graduate School of Medicine, Tohoku University, Sendai, Japan; ^11^Molecular Pathology, Tokyo Medical University, Tokyo, Japan

**Keywords:** cesium (Cs)-137, feces, gut microbiome, cattle, ruminants, elimination, Fukushima Daiichi Nuclear Power Plant

## Abstract

In March 2011, an accident at the Fukushima Daiichi Nuclear Power Plant led to major problems, including the release of radionuclides such as Cesium (Cs)-137 into the environment. Ever since this accident, Cs-137 in foods has become a serious problem. In this study, we determined the concentration of Cs-137 in the feces, urine, and ruminal contents of cattle and demonstrated the possibility of its elimination from the body by intestinal bacteria. The results revealed a high Cs-137 concentration in the feces; in fact, this concentration was higher than that in skeletal muscles and other samples from several animals. Furthermore, intestinal bacteria were able to trap Cs-137, showing an uptake ratio within the range of 38–81% *in vitro*. This uptake appeared to be mediated through the sodium–potassium (Na^+^-K^+^) ion pump in the bacterial cell membrane. This inference was drawn based on the fact that the uptake ratio of Cs-137 was decreased in media with high potassium concentration. In addition, it was demonstrated that intestinal bacteria hindered the trapping of Cs-137 by the animal. Cattle feces showed high concentration of Cs-137 and intestinal bacteria trapped Cs-137. This study is the first report showing that intestinal bacteria contribute to the elimination of Cs-137 from the body.

## Introduction

On March 11, 2011, the Pacific coast of Tohoku was hit by a gigantic earthquake, often referred to as the Great East-Japan Earthquake. This triggered a tsunami that seriously damaged the Tohoku region of northeastern Japan (1). In particular, the Fukushima Daiichi Nuclear Power Plant (FNPP), located on the coastal area, was struck by the tsunami, resulting in one of the worst nuclear accidents at a power plant, followed by widespread fall-out by various radionuclides (2–5).

After the FNPP accident, an evacuation zone was set up within a 20-km radius from the power plant. Many of the local population were forced to take refuge in unaffected areas and had to live in unfamiliar places. However, ~3,400 head of cattle, 31,500 pigs, and 630,000 chickens were left behind in the area (6). On April 22, 2011, the Government of Japan ordered the Fukushima prefectural government to euthanize livestock within the evacuation zone, preventing people from eating meat with radionuclides. Meanwhile, outside this zone, radioactive Cesium (Cs) was detected in foods at concentrations exceeding the reference limit. Therefore, food shipments from parts of Fukushima Prefecture were restricted to allay concerns about foods with radionuclide (7, 8).

Many research papers have mentioned the impact of the FNPP accident and the internal exposure (9–13). Our group reported the distribution of radioactive substances in abandoned cattle, revealing that the highest distribution of Cs-137 was in the skeletal muscle (6). Furthermore, we found that some radionuclides showed organ-specific distribution, such as in the liver, blood, and kidneys (6).

Understanding the Cs-137 distribution is important for evaluation of food safety and to study the biological effects due to the exposure to radioactive substances. The dynamics of Cs-137 in the body are being revealed only gradually. It was previously thought that the major routes of Cs-137 excretion in humans are through urine and feces (14). In addition, livestock excrete Cs-137 via their milk (7). In the intestinal tract, the uptake of inorganic substances takes place against an electrochemical potential difference (15–17). Moreover, the amount of inorganic substances in the intestinal tract differs based on the dietary habits (16). In this study, we postulated that the fecal route is as important as the urinary system for excreting Cs-137. Therefore, we decided to examine the contribution of intestinal bacteria to Cs-137 excretion.

In the intestinal tract, Cs-137 encounters up to 10^14^ bacteria in the mammalian intestine (18). We postulated that the process of Cs-137 uptake was mediated through the metabolic system of the intestinal bacteria. Bacteria transport ions and metabolic products, through channel and membrane transport proteins that exist on their cell surface. These proteins maintain the intracellular conditions of the bacterial cell. However, potassium (K) ion channel does not transport sodium (Na) ions, despite having ion radius larger than that of Na and both ions belong to the same family of elements. The K channel acts as an ion selectivity filter, transporting only K^+^ (19). However, it has been reported, that Cs ions can enter cells through the Na–K^+^ pump (14). The rate of Cs transport is no more than ~0.25 times that of K. Furthermore, estimates of the relative selectivity of K and Cs by the K channels and Na pump have been described (14), with most studies reporting that the Cs:K selectivity ratio varies from <0.02 to ~0.2. Moreover, the typical Cs:K selectivity ratio for the Na pump is 0.25 (14). It is thus clear, that cells transport Cs ion. There have been some publications discussing uptake of Cs by microorganisms as well as proposed mechanisms (20–23). Therefore, we hypothesized that intestinal bacteria can also take up Cs-137 like other bacteria.

In this study, we used *Bifidobacterium, Bacteroides*, and *Clostridium* species for the Cs-137 uptake assays. These species were selected as they are dominant in the bovine intestine (24). We investigated whether feces were associated with Cs-137 elimination from the bodies, and whether intestinal bacteria indeed take up the radionuclide. We also checked for any competitive uptake between Cs-137 and K. These examinations should help in clarifying the contribution of feces in Cs-137 elimination.

## Materials and methods

### Samples

During the period between October 20, 2011 and March 6, 2012, we sampled the skeletal muscle (longissimus muscle) from a total of 23 cattle (15 female and 8 male) in Kawauchi village and Tomioka town. We also obtained the fecal samples from 6 in Kawauchi village and 1 in Tomioka town. Urine was obtained from 3 cattle. In Tomioka town, feces, stomach content, and muscles were obtained from Inobuta (mixed kind; pig and wild boar) samples. Boar–pig hybrids are the hybridized offspring of a cross between the wild boar (*Sus scrofa*) and any domestic pig (*Sus scrofa domesticus*). Inobuta meat is known as a healthy alternative to other main meat products because it is tasty and low in fat. The number of wild Inobuta was increased in Fukushima after the Great East Japan Earthquake.

It has been reported that the highest distribution of Cs-137 is in the skeletal muscle (6). Therefore, by comparing the Cs-137 concentrations in the skeletal muscle and feces, the distribution of the radionuclide in the feces can be determined. In this experiment, we examined if Cs-137 was discharged via the feces and urine in the Fukushima cattle. In order to consider the discharge route, we compared the Cs-137 concentrations in the feces and urine. Furthermore, we assumed that the Cs-137 concentration in ruminal digests would fluctuate due to the digestive processes. Therefore, examination of the Cs-137 concentration at an intermediate point between food intake and excretion of feces was essential. For this purpose, the Cs-137 concentration in the ruminal content was also determined. In addition, we suspected that through the Cs-137 uptake activity, the intestinal flora might play an important role in inhibiting the body's absorption of the radionuclide from the intestine.

Soil and grass samples were collected at the place where the cattle were caught. Soil samples were taken in a square 30 × 30 cm from the surface to the depth of 10 cm. Radioactivity concentration was calculated into kBq/m^2^ by the method previously described (6). Only the leafy portions of grasses were sampled and analyzed.

**Figure 1:**
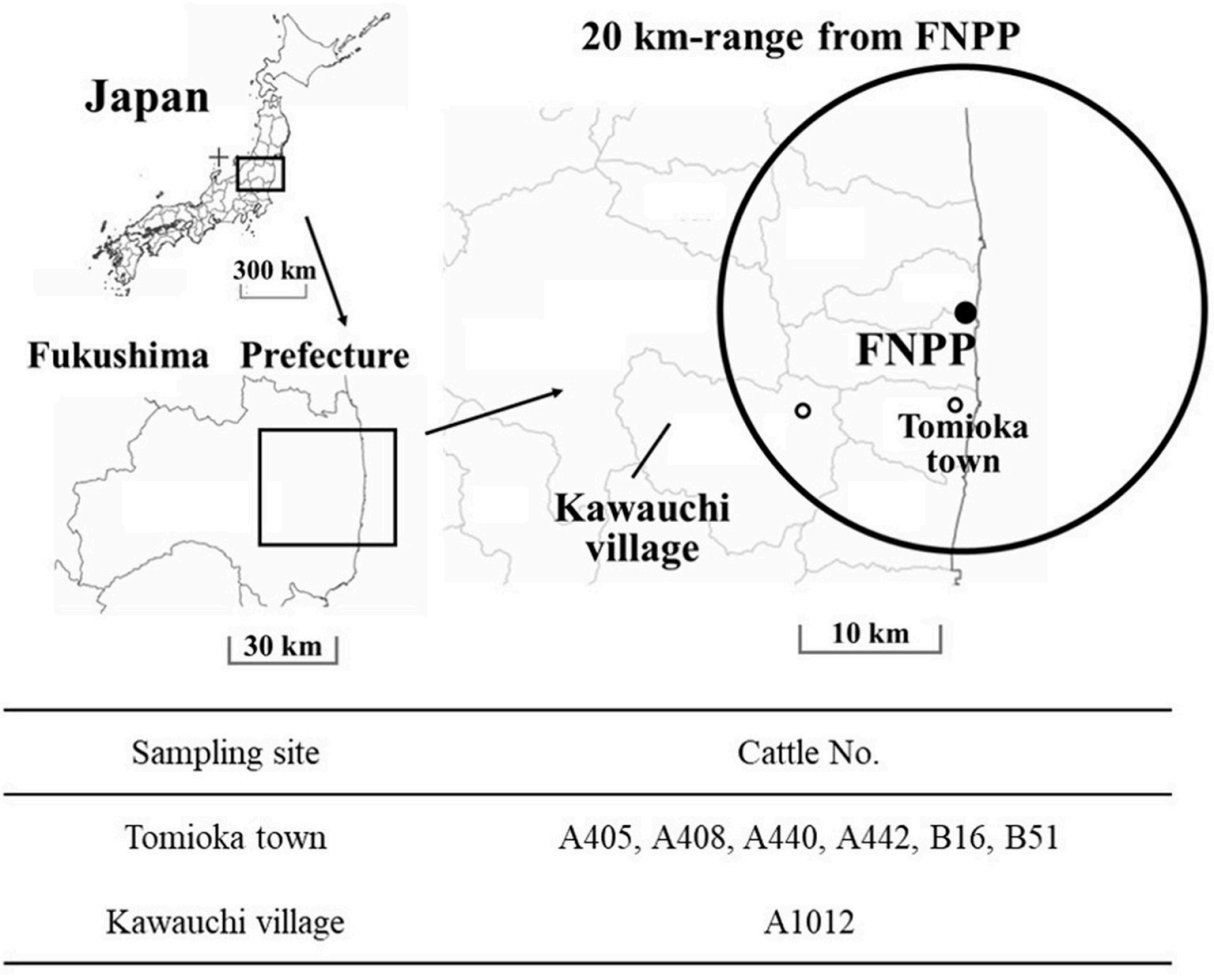
Study sites. Sites of cattle sampling in Fukushima Prefecture, within a range of 20-km from the Fukushima Daiichi Nuclear Power Plant (FNPP). ◦: The locations of sampled cattle, Tomioka town, and Kawauchi village. These locations had different air dose rates. The maps were prepared from open-access base maps freely available for public and academic use (source: http://maps.gsi.go.jp, from the Geographic Information Authority of Japan).

Kawauchi village and Tomioka town represented two different contamination levels of radionuclides (Figure 1). Air dose rate in Kawauchi village was more than 1.0 μSv/h, and 9.5 μSv/h and fewer at sampling time, while that of Tomioka town was more than 3.8 μSv/h, and 19.0 μSv/h and fewer at sampling time, respectively (URL: https://ramap.jmc.or.jp/map/#lat=37.457027049337896&lon=140.83407992880714&z=11&b=std&t=undefined&s=25,0,0,0&c$=$20110429_dr). The cattle demographics are presented in Table 1. We have tried to examine the excretion route of Cs-137 and measure the radioactivity in samples.

**Table 1:** Demographics of the sampled cattle.

**Cattle No**.	**Sampling sites**	**Sampled date**	**Birth**	**Gender**	**Age (months)**	**Ear tag numbers**
A405	K	Nov. 29, 2011	Jul. 5, 2010	F	16	1253570241
A408	K	Dec. 9, 2011	Sep. 1, 2007	F	50	463501274
A440	K	Nov. 29, 2011	Jan. 29, 2010	F	10	⋇
A442	K	Nov. 29, 2011	Aug. 1, 2007	F	51	0240672852
A1012	T	Mar. 6, 2012	Aug. 8, 2010	M	19	1335275224
B16	K	Oct, 20. 2011	Oct. 19, 2010	M	12	1335275323
B51	K	Oct. 20, 2011	Aug. 8, 2010	F	14	1235270289

*Cattle No: Cattle were numbered to adjust the data. Sampling sites: K and T represent Kawauchi village and Tomioka city, respectively. Sampled date and Birth: All cattle had identifying ear tags with unique 10-digit numbers indicating their date of birth. Age: The age of the animal at euthanization. ⋇ indicates that A440 was not provided with ear tag numbers because it was born from A442*.

### Cs-137 Determination in Cattle's Organ

The radioactivity in the bovine skeletal muscle, ruminal contents, urine, and feces were measured with a gamma-ray spectrometer, using a high-purity Germanium detector (Ortec Co., Oak Ridge, TN, USA), as described in our previous study (6, 12). Feces and urine were sampled directly from rectum and bladder of euthanized cattle.

### Bacterial Strains, Media, and Cultures

*Bifidobacterium longum* subsp. *longum* JCM 1217, *Clostridium perfringens* JCM 1290, *Clostridium ramosum* JCM 1298, *Bacteroides fragilis* RIMD0230001, and *Bacteroides vulgatus* JCM 5826 were used as the major intestinal bacteria (25–31). These bacterial strains were propagated in 10 mL of Brain-Heart Infusion (BHI) broth (Difco Laboratories, Detroit, MI, USA) or Gifu Anaerobic Medium (GAM) broth (Nissui Pharmaceutical Co., Tokyo, Japan) using 1% (v/v) inoculums. BHI medium was used for incubation of *B. longum, C. perfringens* and *C. ramosum*, and GAM medium was used for *B. fragilis* and *B. vulgatus* incubation. All bacteria were incubated anaerobically in Anaero-Pack systems (Mitsubishi Gas Chemical, Tokyo, Japan) at 37°C for 24 h. The BHI and GAM broths were sterilized at 121°C for 15 min and 115°C for 15 min, respectively.

### Cs-137 Determination in Bacteria

Muscles were sampled from the cattle living within 20-km range from the FNPP and muscle extract was prepared by boiling. BHI agar and GAM agar containing 10% (v/v) of the Cs-137-containing extract were used as the incubation media for the bacterial Cs-137 uptake assay. The number of viable bacteria was adjusted to 10^8^ colony forming units (CFUs)/ml, and 100 μl of each bacterial strain was inoculated into the respective incubation medium and incubated at 37°C for 48 h under anaerobic conditions. After the incubation, the media were washed three times using 1 ml of sterile Dulbecco's phosphate-buffered saline (PBS) (Nissui Pharmaceutical Co., Tokyo, Japan). The bacterial suspension was recovered and poured into U8 (100 mL) polypropylene containers (Yamayu, Osaka, Japan). The agar medium was also melted and poured into a separate U8 polypropylene container. The agar medium from the three Petri dishes was poured into U8 containers. The concentration of radioactive Cs in the bacterial cells and media was detected using Germanium gamma-ray spectrometry. Results of radioactivity in several organs were expressed as Bq/fresh weight. Furthermore, blank test was conducted to examine how Cs-137 was extracted by washing. Blank test was done using the same protocol without inoculating it with bacteria. Risk assessment was performed for the handling of radioactive substances among the people involved. In addition, when we measured the sample's radiation using survey meter, results were within the background levels.

### Inhibition of Cs-137 Uptake

To examine the Cs-137 uptake inhibition caused by K^+^ in the medium, K_2_HPO_4_ was added to BHI and GAM agar at a final concentration of 1,500 ppm. After cultivation under anaerobic conditions, the concentration of Cs-137 was detected in both bacterial cells and the media as described above. To confirm the K^+^ concentration in the BHI and GAM medium, a LAQUA Twin Compact Water Quality Meter (HORIBA Ltd., Kyoto, Japan) was used according to the provided protocols.

### Statistical Analysis

Differences of Cs-137 concentration between female and male were examined by Student *t-*test. Differences of Cs-137 concentration among feces, skeletal muscle and stomach contents were calculated by Turkey-Kramer test. Differences in the uptake ratio of Cs-137 in media with or without added K were analyzed with the two-way analysis of variance (ANOVA). Furthermore, significant differences among the strains were calculated with the Turkey-Kramer test. Significant differences between media with K and without K were calculated with Student *t*-test. Probability values of *p*<0.05 were considered significant. Each sample was measured in triplicate.

## Results

### Detection of Cs-137 in the Skeletal Muscle, Feces, Urine, and Ruminal Contents

The concentration of Cs-137 in the skeletal muscle (Bq/kg) was 639.1 (female, *n* = 10) and 536.8 (male, n = 5) in Kawauchi village, and 2705.4 (female, *n* = 5) and 2962.9 (male, *n* = 3) in Tomioka town. There was no significant difference in Cs-137 concentration in male and female animals in the two geographic regions. In eight of these animals, fecal samples were also taken, and in some of these, urine and ruminal samples were also obtained. The respective concentrations of Cs-137 in these animals are shown in Table 2. The Cs-137 concentration in the skeletal muscle was higher than that in the feces for some samples. However, the Cs-137 concentration in the feces from A1012 was 9.5 times higher than that in the skeletal muscle. A1012 was located in Tomioka town, a highly contaminated area located at a distance of 3 km from FNPP. Likewise, the Cs-137 concentration in the feces from B51 (located 5 km from FNNP) was 4.6 times higher than that in the skeletal muscle. In this study, we obtained only three samples in feces, urine and ruminal contents because condition of samples was different. In particular, urine and ruminal contents were not contained in the bladder and rumen, at the time of sampling. A high deposition of Cs-137 was observed in the feces and this was higher than that in the ruminal contents and urine (Table 2). The Cs-137 concentration was 2–53 times higher in the feces than in the ruminal contents. In addition, cattle housed in non-contaminated areas did not have detectable Cs-137 levels (6). Radioactivity Cs-137 concentration was about 2,300 and 2,700 Bq/kg in the soil open area, and 3,000 and 3,700 Bq/kg in grass (Japanese pampas grass) with grazed marks in Kawauchi village. In Inobuta sampled in Tomioka town, feces were significantly higher than skeletal muscle and stomach contents (Table 3, *p* < 0.01).

**Table 2:** Distribution of Cs-137 in skeletal muscle, feces, urine, and 1st stomach contents.

**Cattle No**.	**Cs-137 (Bq/kg)**			
	**Skeletal muscle**	**Feces**	**Urine**	**Ruminal contents**
A405	604.2 ± 10.3	226.1 ± 6.2	98.8 ± 3.4	101.7 ± 1.9
A408	669.5 ± 13.9	419.0 ± 6.0	N. D.	N. D.
A440	748.4 ± 16.5	494.6 ± 9.9	N. D.	N. D.
A442	663.7 ± 14.9	167.4 ± 4.0	N. D.	N. D.
A1012	2447.9 ± 48.1	23213.5 ± 172.5	N. D.	N. D.
B16	635.0 ± 23.9	213.0 ± 13.0	169.5 ± 3.5	85.1 ± 1.4
B51	414.6 ± 18.0	1887.1 ± 17.1	35.3 ± 1.3	35.3 ± 3.6

*Cattle No.: The individual identification number for each animal. Data are presented as the mean ± SD. N. D., Non data*.

**Table 3:** Cs-137 concentration in Inobuta.

**Cs-137 concentration (Bq/kg)**	
Skeletal muscle	1100.0 ± 200.0^*^
Feces	5464.3 ± 2923.8
Stomach contents	996.0 ± 648.6^*^

*Concentration of Cs-137 are shown in mean ± standard deviations*.

*N. D, Non data*.

* *Significantly different from the feces. Significant difference at the 99% confidence level, using the Tukey-Kramer test*.

### Uptake of Cs-137 by Intestinal Bacteria

To examine Cs-137 uptake by intestinal bacteria, we chose to use common bacterial strains found in the bovine intestine. (24). Cs-137 was detected from both the bacterial suspension and the medium (Table 4). The uptake ratio was calculated as the radioactivity in the bacterial suspension divided by the total radioactivity (bacterial suspension plus medium) and multiplied by 100. It was observed that the bacterial suspension had a higher Cs-137 dose than the medium. Although each bacterium took up Cs-137, the uptake ratio was different among different species, with *B. vulgatus* showing the highest value. The significant differences have been presented in Table 4. In the results of blank test, the amount of Cs-137 extracted by water was less compared to the bacterial uptake.

**Table 4:** Uptake of Cs-137 by intestinal bacteria.

**Group**	**Cs-137 concentration; BHI (×10^3^ nBq/mg)**			**Cs-137 concentration; BHI added K_2_HPO_4_ (×10^3^ nBq/mg)**		
	**Bacteria**	**Medium eliminated bacteria**	**Uptake ratio (%)**	**Bacteria**	**Medium eliminated bacteria**	**Uptake ratio (%)**
*B. longum*	5.98 ± 1.4	9.84 ± 0.8	37.8 ± 3.1^b^	159.5 ± 4.2	108.4 ± 2.8	59.5 ± 0.4^a*^
*C. perfringens*	9.92 ± 2.0	12.1 ± 3.6	45.0 ± 2.8^b^	27.3 ± 1.6	103.1 ± 3.4	21.0 ± 0.6^b*^
*C. ramosum*	9.46 ± 1.0	3.19 ± 1.5	74.8 ± 1.1^a^	28.9 ± 1.3	102.3 ± 3.5	22.0 ± 0.5^bc*^
*B. fragilis*	9.26 ± 1.0	3.80 ± 1.6	70.9 ± 1.2^a^	32.7 ± 1.9	100.9 ± 3.1	24.5 ± 0.6^c*^
*B. vulgatus*	11.0 ± 1.4	2.55 ± 1.9	81.2 ± 1.2^a^	29.6 ± 1.6	103.1 ± 3.4	22.3 ± 0.4^bc*^
Medium only	2.7 ± 0.1	105.5 ± 9.7	2.6 ± 0.1	3.8 ± 0.4	110.7 ± 3.6	3.4 ± 0.3

*Data are presented as the mean ± SD. Statistical significances were calculated by two-way analysis of variance (Strains × Concentration). The main effect for both Strains and Concentration and the interaction are significant [Strains F _(4, 20)_ = 37.10, p < 0.01, Concentration F _(1, 20)_ = 1094.37, p < 0.01, Interaction F _(4, 20)_ = 243.10, p < 0.01]*.

* *Show the significant differences compared with group of media without added potassium (p < 0.01). Significant differences were calculated with Student t-test. a, b, c. The same letters represent the no significant differences at 99% confidence level, compared among five bacterial strains in the groups of media with or without added K, respectively. Significant differences were calculated with Turkey-Kramer test*.

### Inhibition of Cs-137 Uptake

The Cs-137 uptake ratio for all the strains, except *B. longum*, was significantly lower in the supplemented medium than in the non-supplemented BHI (*p* < 0.01) (Table 4). In contrast, *B. longum* showed an increase of Cs uptake after the addition of K. The K^+^ concentration were about 1,500 ppm (Table 5).

**Table 5:** Confirmation of K^+^ concentration.

**Medium**	**K^+^ concentration (ppm)**
BHI	223.3 ± 3.3
GAM	903.3 ± 3.3
BHI added K_2_HPO_4_	1566.7 ± 33.3
GAM added K_2_HPO_4_	1466.7 ± 33.3

*Data are presented as the mean ± SD*.

## Discussion

Obtained results showed that activity concentration in feces were higher than in muscle. Therefore, the excretion of Cs-137 via feces is important for discussion on the dynamics of radionuclide uptake in the body. In addition, bacterial uptake of Cs-137 was examined in this study. This uptake model showed that intestinal bacteria take up Cs-137. It is well-known that there are a huge number of viable bacteria in feces. It was reported that bacterial biomass is a major component of organic substances in the feces (32). Therefore, it is possible that the uptake of Cs-137 by intestinal bacteria is related to its high distribution in the feces.

It has been demonstrated that the concentration of Cs-137 in feces is higher than ruminal contents (Table 2). This result was obvious because the bulk of the ruminal contents will lighten through the digestive process. As a result, the concentration of Cs-137 in the ruminal contents would be lower. Furthermore, it is possible that Cs-137 in the ruminal contents is transferred to the blood in the process of digestion. Moreover, it is also likely that this result was caused by the intestinal bacteria taking up Cs-137, which was then secreted to the intestinal tract and subsequently excreted through the feces. It does not have to consider the possibility of returning Cs-137 to the intestine via bile. Leggett et al. showed that biliary secretion represents only a few percent of the total percent of Cs-137 in liver (14). In conclusion, it is obvious that the fecal route is the most important pathway of Cs-137 elimination from cattle body. As seen in Table 2, the concentration of Cs-137 in the feces was higher than that in urine, again showing the contribution of fecal discharge of the radionuclide. In this study, we specifically collected samples from the Japanese black breed, which generally discharges daily about 30 and 20 kg of feces and urine, respectively (Unpublished data). The amount of discharge in livestock fluctuates according to body weight, types of livestock and feed, and breeding form, among other parameters. Generally, these values are used in the scale calculations of feces and urine processing facilities. Therefore, it was thought that the amount of discharge and the Cs-137 concentration in feces are higher than that of urine, indicating that feces eliminate Cs-137 from the body more efficiently than urine. In a previous study, urine was concluded as the main route for Cs-137 discharge from the body because of its water-soluble characteristics (14). In a previous study, urine was concluded as the main route for Cs-137 discharge from the body because of its chemical characteristics, which is in contrast with these results. This suggest that feces have a higher contribution than urine in this regard.

In this study, we examined the radioactivity in skeletal muscle and feces. Radioactive Cs concentration in organs is dependent on the feeding conditions and the geographic location of cattle (33). Cattle used for sampling were born before the FNPP accident occurred. These cattle have been exposed radioactive substances until they are euthanized. Furthermore, in our previous study we have showed the radioactivity concentration of Cs in the soil and grass (6). In Tomioka town, Cs-137 concentration was 10,000 ~ 25,000 Bq/kg in the soil (http://www.maff.go.jp/j/wpaper/w_maff/h23_h/trend/part1/sp/sp_c2_2_02.html) and 1,000 ~ 10,000 Bq/kg in the grass (Unpublish data), respectively. The Cs-137 concentration in soil and grass in Tomioka town was higher than that in Kawauchi village. The Cs-137 concentration of grass was found have high radioactivity concentration and this was eaten by the cattle in dairy. Therefore, it was thought that radioactive Cs was accumulated in cattle body. In addition, Cs-137 concentration of feces was higher than skeletal muscle in Inobuta (mixed kind; pig and wild boar) (Table 3). High distribution of Cs-137 was also shown monogastric animals and in Tomioka town. Moreover, it has been reported that the fecal route is an important route in other ruminants (lambs and ewes) and shown to be approximately equal to urinary excretion for radioactive Cs (34). The study of this paper revealed that concentration of Cs-137 in feces was higher than in muscle and urine. Therefore, the results of this paper are consistent with previous studies. Furthermore, it was suggested that feces contribute to the excretion of Cs-137 in ruminants.

It was thought that intestinal bacteria were able to trap Cs-137 and the uptake ratio was different among the species (Table 3). Thus, even though intestinal bacteria are related to the intake and discharge of Cs-137, their uptake ratio differs depending on the strain type. The uptake of Cs-137 with K occurs through the K^+^ pump located in the bacterial cell membrane (14). The element Cs is homologous to K, and hence, both exhibit similar behavior. Therefore, bacteria take up Cs-137 in the same way they do for K. The *Bacteroides* species and *C. ramosum* showed high uptake ratios, whereas that of *B. longum* was low. The reason for this result is unclear, but this could be related to the structure of the bacterial surface layers since *Bacteroides* species are Gram-negative, whereas *Clostridium* and *Bifidobacterium* species are Gram-positive. In addition, it was reported by Kato et al. (35), that *Bacteroidetes* bacteria, especially *Flavobacterium* spp. appear to have significant tolerance to high concentrations of Cs^+^ *in vitro*. Therefore, in this result, it was thought that *B. fragilis* and *B. vulgatus* were able to accumulate Cs-137 actively. Moreover, because Cs is homologous to K, the requirement of K in the metabolic system could be related to the uptake of Cs-137. More examination is needed to clarify the reasons behind these results.

The Cs-137 uptake ratio by some strains was significantly lower in the supplemented medium than in the non-supplemented BHI (Table 4). These results indicated that the uptake of Cs-137 could be inhibited in most strains by increase of the K concentration in the medium. In addition, the growth in the supplemented medium showed no significant difference compared with the non-supplemented medium (data not shown). This further suggests that the uptake of Cs-137 is related to that of K. Since the uptake of Cs-137 is inhibited by K, contamination by Cs-137 depends on the K concentration in the body. In another study, Cs-137 uptake was also found to be inhibited by K^+^s in soil microbes in a dose-dependent manner (31). It is interesting to note that the Cs-137 uptake by *B. longum* significantly increased due to the addition of K; the reason for this remains to be elucidated.

It is thought that potassium ion is an essential element for bacteria. Actually, K is required for the activity of the ribosome and a lot of enzymes (36). However, it has not been reported the relationship between intestinal bacteria and potassium ion. Recent study, *Lactobacillus rhamnosus* JB-1 demonstrated that K^+^ uptake in the intestine contribute to the beneficial effect for allergic and other inflammatory disorders (37). This study suggested that K^+^ uptake contribute to the health of host. In this study, we demonstrated that intestinal bacteria contribute to Cs-137 excretion for host. K^+^ transporter help to excrete Cs-137. It was suggested that K^+^ transporter of intestinal bacteria also show beneficial effects.

In conclusion, we demonstrated that intestinal bacteria contribute to the elimination of Cs-137 from the body of cattle. The data of this study provides little information because it was in a limited condition, hence larger studies with higher number of animals will be required to make this statement in the further study. During the digestive process, Cs-137 was not only absorbed in blood but also taken up by intestinal bacteria and subsequently discharged via the fecal route. However, there is also a possibility that intestinal bacteria maintain Cs-137 in the intestinal tract. It is important to consider that many factors influence uptake of Cs^+^ *in vivo*. As a result, feces contribute to the excreting of Cs-137 from cattle. In future studies, we plan to examine the mechanism of Cs-137 elimination in detail and the possibility of eliminating this radionuclide using other bacteria.

## Data Availability

The datasets generated for this study are available on request to the corresponding author.

## Ethics Statement

Each experimental protocol was approved by the Institutional Ethics Commissions for Animal Research at Tohoku University. Our collection of organs from the euthanized cattle was in collaboration with the units of veterinary doctors of both the Livestock Hygiene Service Centre (LHSC) of Fukushima Prefecture and the Ministry of Agriculture, Forestry and Fisheries, Japan. The veterinary doctors of the LHSC euthanized the cattle in strict accordance to procedures laid out by the Regulation for Animal Experiments and Related Activities at Tohoku University (Regulation No. 122). The cattle owners could be identified on the ear tag of each animal, and their informed consent was obtained by the veterinary doctors of Fukushima Prefecture.

## Author Contributions

KS, KK, RS, YK, TS, HS, HidY, TF, JK, YA, JN, YU, HirY, MF, and EI performed the experiments (e.g., sample collection, measurement of the radionuclide activity in the tissues, bacterial experiments. KS and EI designed the research. KS, JN, YK, and EI wrote the manuscript.

### Conflict of Interest Statement

The authors declare that the research was conducted in the absence of any commercial or financial relationships that could be construed as a potential conflict of interest.

## Abbreviations

FNPP: Fukushima Daiichi Nuclear Power Plant.
